# Announcement: 5th Chemical Congress of North America. Symposium: Chemistry, Biochemistry and Therapeutic Applications of Vanadium

**DOI:** 10.1155/MBD.1996.162

**Published:** 1996


					5TH CHEMICAL CONGRESS
OF NORTH AMERICA
11-15 November, 1997 Cancun, Quintana Roo, Mexico
Sy.mposium: Chemistry, Biochemistry
and Tlierapeutic Applications of Vanadium
Organisers" Debbie C. Crans and Alan S. Tracey
The chemistry section of the symposium will concentrate on. the chemistry qf vanadium that
.has .possible relevance to vanadium b_ioche.mistry or therapeutic applications.. The
biochemistry section will cover topics trom the essentiality of vanadlpm to vanadium-
dependent haloperoxidases to vanadium bioactivity in cell cuItures. The therapeutic section
will predominantly, address the insulin-mimetic properties of vanadium.
The symposium will occur over a time period of' 1 1/2 days.
There will be 14 .speaker.s. fro.m Europe, Japan, the Americas and the Middle East. In
addition there will be contrilguted talks and an accompanying poster session.
A prelimin_a programme is given below:
Alison Butler, Santa Barbara, California, USA
Vanadium bromoperoxidase: enzyme _and functional mimetic studies
Anna M. Cortizo & Susana B. Eteheverry, La Plata, Republica Argentina
Vanadium bioactiviron cells in culture.
Debbie C. Crans, Fort Collins, Colorado, USA
Phosphatase inhibition by vanadium compounds: consideration of aqueous vanadium chemistry
C. lonald Kahn, Boston, Massachusetts, USA
Mechanisms and role of vanadium treatment in diabetes mellitus
Marvin W. Makinen, Chicago, Illinois, USA
Structure and coordination environment ofvanadyl (VO2+) in proteins and enzymes
Forrest H. Nielsen, Grand Forks North Dakota, USA
The nutritional essentiality and physiological metabolism of vanadium in higher animals
Chris Orvig, Vancouver, Britlsti Colunbia, Canad.a
_Coordination chemistry of insulin-mimetic compounds
_Vincent Lo Peeoraro, Ann Arbor, Michig.an, USA
lunctional models for vanadium haloeroxiases
Barry Io Posner, Montreal, Quebec;Canada
The insulin receptor-associated-phosrhotyrosine phosphatase
Lae Pettersson, Umea, Sweden
S_tu-dies of HVO/organic ligand systems using potentiometry and NMR spectroscopy
_Dieter Rehder,Hafnburg, Germany.
Structural and functional models for 15iogenic vanadium compounds
Htiromu
Sakurai, .Kyoto, Japan
ructure-activity relat0nships of the insulin-mimetic vanadyl complexes
Yoram Shech/er, Rehovot, Israel
On the mechanisms of action of vanadium in facilitating its insulin-like effects" the involvement
of protein phosphotyrosine p.hosphatases and nonreceptor protein tyrosine kinases
Alan S. Tracey, Burnaby, British Columbia, Canada
The role of vanadium as a transition-state analogue to phosphate transter in enzymatic reactions
Contact: Alan S. Tracey
Department of Chemistry, Simon Fraser University
Burnaby BC V5A 1S6 CANADA
Email: tracey@sfu.ca
162

				

